# Expression of Wnt-signaling pathway genes and their associations with miRNAs in colorectal cancer

**DOI:** 10.18632/oncotarget.23636

**Published:** 2017-12-23

**Authors:** Martha L. Slattery, Lila E. Mullany, Lori C. Sakoda, Wade S. Samowitz, Roger K. Wolff, John R. Stevens, Jennifer S. Herrick

**Affiliations:** ^1^ Department of Medicine, University of Utah, Salt Lake City, Utah, USA; ^2^ Division of Research, Kaiser Permanente Northern California, Oakland, California, USA; ^3^ Department of Pathology, University of Utah, Salt Lake City, Utah, USA; ^4^ Department of Mathematics and Statistics, Utah State University, Logan, Utah, USA

**Keywords:** wnt-signaling, colorectal cancer, miRNA, gene expression

## Abstract

The Wnt-signaling pathway functions in regulating cell growth and thus is involved in the carcinogenic process of several cancers, including colorectal cancer. We tested the hypothesis that multiple genes in this signaling pathway are dysregulated and that miRNAs are associated with these dysregulated genes. We used data from 217 colorectal cancer (CRC) cases to evaluate differences in Wnt-signaling pathway gene expression between paired CRC and normal mucosa and identify miRNAs that are associated with these genes. Gene expression data from RNA-Seq and miRNA expression data from Agilent Human miRNA Microarray V19.0 were analyzed. We focused on genes most strongly associated with CRC (fold change (FC) of >1.5 or <0.67) and that were statistically significant after adjustment for multiple comparisons. Of the 138 Wnt-signaling pathway genes examined, 27 were significantly down-regulated (FC<0.67) and 32 genes were significantly up-regulated (FC>1.50) after adjusting for multiple comparisons. Thirteen of the 66 Wnt-signaling genes that were differentially expressed in CRC tumors were associated with differential expression of miRNAs. A total of 93 miRNA:mRNA associations were detected for these 13 genes. Of these 93 associations, 36 miRNA seed-region matches were observed, suggesting that miRNAs have both direct and indirect effects on Wnt-signaling pathway genes. In summary, our data supports the hypothesis that the Wnt-signaling pathway is dysregulated in CRC and suggest that miRNAs may importantly influence that dysregulation.

## INTRODUCTION

The Wnt-signaling pathway is an important signaling pathway in many types of cancer including colorectal cancer (CRC) [1]. The canonical Wnt-signaling pathway, and the one studied the most with CRC, is mediated via Wnt ligands and their receptors resulting in accumulation of β-catenin [2]. Components of this pathway include the adenomatous polyposis coli (*APC*) gene which is mutated in roughly 80% of CRC, *AXIN* 1 and 2, and glycogen synthase kinase 3β (*GSK-3β*) [3]. Downstream genes in this pathway include c-*myc*, c-*jun*, and Cyclin D1 (*CCND1*). Two non-canonical Wnt-signaling pathways, Wnt/CA2+ and Wnt/planar cell polarity (PCP), also exist [4]. While these Wnt-signaling pathways have been studied less thoroughly than the canonical Wnt/β-catenin pathway, it is felt that the Wnt-signaling pathways do not operate independently of each other. For instance both *CaMKII* and *NFAT* in the Wnt/CA2+ pathway influence β-catenin [5]; β-catenin has been linked to *JNK* in the Wnt/PCP pathway [6]; and drug response may be influenced by both the canonical and non-canonical pathways [7].

Genomic abnormalities in the Wnt-signaling pathway are a component of CRC tumorigenesis. MicroRNAs (miRNA) can directly influence gene expression by binding to the 3′un-translated region of the target mRNA and promoting mRNA degradation and/or inhibit mRNA translation; imperfect base pairing between the miRNA and mRNA can also result in translation repression of the target gene translation [8, 9]. Associations of miRNAs with targeted genes (TG) can be through direct binding to genes or through a pathway effect where down-stream regulation of gene expression from feedback and feedforward loops occur [10]. Several miRNAs, including miR-34, miR-320, miR-200, and Let-7, have been reported as being associated with Wnt-signaling pathway genes [2]. Additionally, miR-34 and miR-25 have been linked to β-catenin in early development [4]. Expression of Wnt-signaling pathway genes, including *CTNBB1* (gene that encodes β-catenin), *MYC*, and *CCND1*, also have been associated with expression of multiple miRNAs [11]. Peng and colleagues in their review of miRNAs and the Wnt/β-catenin signaling pathway in cancer, provides additional support for the inter-relationship of these factors [12].

In this study we identified genes in the Wnt-signaling pathway using the human Kyoto Encyclopedia of Genes and Genomes (KEGG) pathway database. We determined which genes in the Wnt-signaling pathway are differentially expressed in CRC and if those differentially expressed genes are associated with miRNA differential expression. To help determine if the association between the miRNA and mRNA was direct or indirect, i.e. through another TG::miRNA that influences pathway genes, we identified seed-region matches between the TG and miRNA. We evaluate if miRNAs are associated with Wnt-signaling pathway genes and if genes in the canonical and non-canonical pathways are associated with the same miRNAs as other genes in the same canonical or non-canonical Wnt-signaling pathways.

## RESULTS

The majority of cases were diagnosed with colon cancer (77.9%) rather than rectal cancer (Table 1). The majority of the study were men (54.4%), non-Hispanic white (74.2%), and had a *TP53*-mutated tumor (47.5%). MSI samples represented 13.4% of the study and MSS samples comprised 86.6% of the study population.

**Table 1:** Description of study population

	*N*	%
Site		
Colon	169	77.9
Rectal	48	22.1
Sex		
Male	118	54.4
Female	99	45.6
Age		
Mean (SD)	64.8	10.1
Race		
non-Hispanic White	161	74.2
Hispanic	14	6.5
non-Hispanic Black	8	3.7
Unknown	34	15.7
AJCC Stage		
1	58	27.1
2	61	28.5
3	72	33.6
4	23	10.8
Tumor Phenotype		
*TP53* mutated	103	47.5
*KRAS* mutated	69	31.8
*BRAF*-mutated	21	10.1
CIMP High	45	20.7
MSI	29	13.4
Vital Status		
Dead	92	42.6
Alive	124	57.4

**Figure 1:**
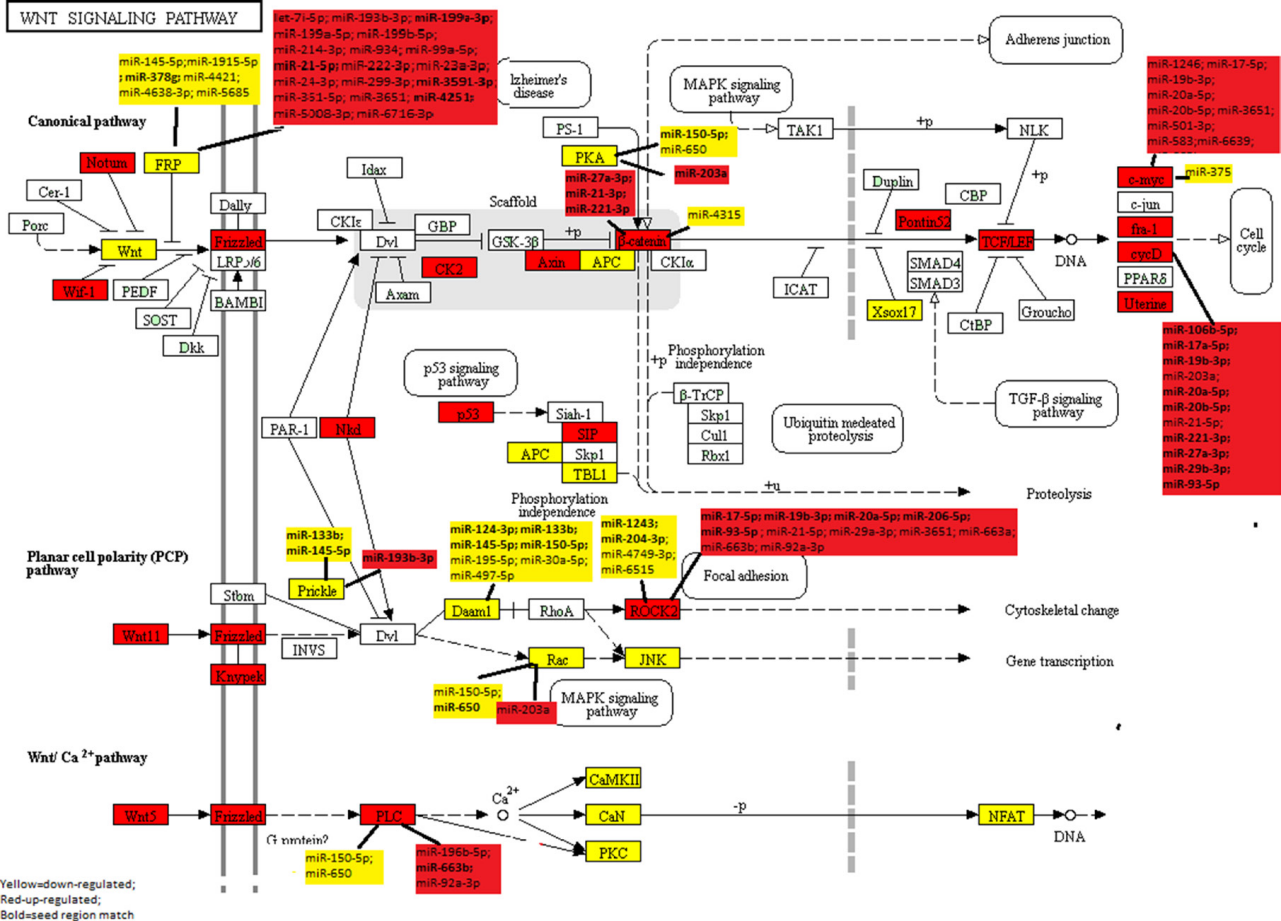
KEGG Wnt-Signaling Pathway: Dysregulated mRNA and miRNA in colorectal cancer

Of the 138 Wnt-signaling pathway genes examined (43.5%), 27 were significantly down-regulated (FC<0.67) and 32 genes were significantly up-regulated (FC>1.50) after adjusting for multiple comparisons when evaluating CRC tumors overall (Supplementary Table 1), this compares to 37.5% of all protein-coding genes being dysregulated (6541 dysregulated of 17461 protein-coding genes). Associations were similar when looking at MSS only, although FC were slightly greater for MSS-specific tumors, the difference was minor given that over 85% of all tumor samples were MSS (Supplementary Table 3). The FC of four genes was altered sufficiently to merit further analysis based on our criteria of <0.67 or >1.50 for MSS tumors versus overall (*APC2* FC_mss_ 0.65, FC_overall_ 0.68; *FZD6* FC_mss_ 1.54, FC_overall_ 1.48; *CSNK2A1* FC_mss_ 1.54, FC_overall_ 1.49; *DKK1* FC_mss_ 1.87; FC_overall_ 1.47). Figure 1 visually illustrates the up-regulated genes (red) and the down-regulated pathway genes (yellow) within the Wnt-signaling pathway.

Further evaluation of significant Wnt-signaling pathway genes with miRNAs showed that 13 of the 66 Wnt-signaling genes that were differentially expressed in CRC tumors overall or specifically with MSI or MSS tumors and were associated with differential expression of miRNAs after adjustment for multiple comparisons (Table 2). Only one gene, *LEF1*, that was statistically significantly associated with two miRNAs, was excluded because the miRNA’s FCs were outside of the 0.67 and 1.50 cutpoints. Of the 13 genes associated with miRNAs, six were down-regulated in carcinoma tissue and seven were up-regulated in carcinoma tissue relative to normal mucosa. All of the genes were associated with multiple miRNAs, with *SFRP5* being associated with 24 miRNAs, *ROCK2* with 15 miRNAs, *MYC* with 13 miRNAs, *CCND1* with 11 miRNAs, and *SFRP4* with 10 miRNAs. While 33 miRNAs were associated with only one gene, many miRNAs were associated with multiple genes: miR-7i-5p, miR-133b, miR-221-3p, miR-27a-3p, miR-29a-3p, miR-6515-5p, miR-663a, and miR-93-5p were associated with two genes; miR-145-5p, miR-17-5p, miR-193-3p, miR-19b-30, miR-203a, miR-20a-5p, miR-21-5p, miR-3651, miR-650, and miR-663b with three genes, and miR-92a-3p, miR-150-5p, and miR-20b-5p with four genes. Figure 1 further illustrates the pathway relationships between the differentially expressed Wnt-Signaling Pathway genes and miRNAs. As shown in Figure 1, several of the miRNAs associated with these genes are downstream of other genes in the same pathway.

**Table 2:** Associations between dysregulated genes in the Wnt-signaling pathway and miRNAs

Gene Name	Tumor Mean	Normal Mean	Fold Change	miRNA^1^	Tumor Mean	Tumor SD	Normal Mean	Normal SD	Fold Change	Beta	Raw *p*-value	FDR *p*-value
*CSNK2A2*	46.72	23.14	2.02	**hsa-miR-20b-5p**	17.65	15.14	3.3	3.59	5.35	0.3	<.0001	0.0271
				hsa-miR-6515-5p	1.2	2.14	4.41	2.47	0.27	–0.31	<.0001	0.0271
				hsa-miR-92a-3p	121.6	104.09	41.18	24.92	2.95	0.29	<.0001	0.0271
*PLCB4*	325.61	166.3	1.96	hsa-miR-196b-5p	17.89	19.62	5.53	5.43	3.24	0.26	0.0003	0.0407
				**hsa-miR-663b**	65.5	24.80	32.21	14.68	2.03	0.22	0.0003	0.0407
				hsa-miR-92a-3p	121.6	104.09	41.18	24.92	2.95	0.26	0.0003	0.0407
*SFRP4*	35.37	5.03	7.03	hsa-let-7i-5p	62.16	37.92	39.97	21.97	1.56	0.26	0.0004	0.0233
				hsa-miR-145-5p	132.97	156.85	223.14	182.21	0.6	0.25	0.0004	0.0233
				**hsa-miR-193b-3p**	9.12	7.78	5.42	3.77	1.68	0.27	0.0002	0.0136
				hsa-miR-199a-3p	44.83	37.37	22.53	15.51	1.99	0.34	<.0001	0.0081
				hsa-miR-199a-5p	20.18	17.86	9.28	6.76	2.17	0.38	<.0001	0.0081
				hsa-miR-199b-5p	4.69	4.05	1.53	1.67	3.07	0.39	<.0001	0.0081
				hsa-miR-214-3p	13.24	10.86	6.13	4.14	2.16	0.35	<.0001	0.0081
				hsa-miR-934	4.36	3.61	0.94	1.36	4.66	0.31	0.0002	0.0136
				hsa-miR-99a-5p	6.3	7.51	3.7	3.82	1.71	0.34	<.0001	0.0081
*CCND1*	317.79	122.64	2.59	**hsa-miR-106b-5p**	15.9	13.81	5.19	4.13	3.06	0.25	0.0006	0.0349
				**hsa-miR-17-5p**	61.04	48.49	16.38	10.13	3.73	0.3	<.0001	0.0244
				**hsa-miR-19b-3p**	29.8	23.72	10.42	9.70	2.86	0.28	0.0003	0.0244
				hsa-miR-203a	12.52	13.86	3.7	4.29	3.38	0.27	0.0002	0.0244
				**hsa-miR-20a-5p**	70.78	59.44	17.61	12.25	4.02	0.28	<.0001	0.0244
				**hsa-miR-20b-5p**	17.65	15.14	3.3	3.59	5.35	0.29	<.0001	0.0244
				hsa-miR-21-5p	463.11	312.01	167.37	118.91	2.77	0.25	0.0006	0.0349
				**hsa-miR-221-3p**	13.53	12.32	4.12	4.07	3.28	0.26	0.0003	0.0244
				**hsa-miR-27a-3p**	56.26	37.77	23.29	14.07	2.42	0.27	0.0002	0.0244
				**hsa-miR-29b-3p**	24.31	22.60	9.83	8.73	2.47	0.27	0.0003	0.0244
				**hsa-miR-93-5p**	41.72	32.63	15.2	9.19	2.74	0.26	0.0002	0.0244
*SFRP5*	0.67	1.23	0.54	hsa-let-7i-5p	62.16	37.92	39.97	21.97	1.56	0.26	0.0019	0.043
				hsa-miR-1915-5p	1.04	1.15	1.77	1.37	0.59	0.22	0.0023	0.0459
				hsa-miR-193b-3p	9.12	7.78	5.42	3.77	1.68	0.22	0.0018	0.0419
				**hsa-miR-21-5p**	463.11	312.01	167.37	118.91	2.77	0.24	0.0038	0.047
				hsa-miR-222-3p	19.45	14.36	11.08	6.44	1.76	0.21	0.0045	0.0488
				hsa-miR-23a-3p	174.68	110.22	87.53	50.12	2	0.31	0.0007	0.038
				hsa-miR-24-3p	106.75	61.76	62.39	29.22	1.71	0.3	0.0015	0.0419
				hsa-miR-29a-3p	110.29	84.95	51.04	29.87	2.16	0.21	0.0041	0.047
				**hsa-miR-3591-3p**	3.34	3.84	1.97	2.43	1.7	-0.27	0.0006	0.038
				hsa-miR-361-5p	11.62	9.05	6.2	3.98	1.87	0.25	0.0005	0.038
				hsa-miR-3651	58.66	34.62	25.92	12.63	2.26	0.27	0.0011	0.0419
				**hsa-miR-378g**	1.19	1.33	2.46	1.56	0.48	0.22	0.0014	0.0419
				**hsa-miR-4251**	3.31	2.10	1.77	1.83	1.86	0.2	0.0038	0.047
				hsa-miR-4421	1.77	1.89	2.95	1.88	0.6	0.24	0.0004	0.038
				hsa-miR-4638-3p	0.78	0.94	1.26	1.09	0.62	0.28	0.0007	0.038
				hsa-miR-5008-3p	2.65	1.88	1.12	1.81	2.38	0.2	0.005	0.0496
				hsa-miR-5685	1.28	1.78	2.78	1.71	0.46	0.23	0.0011	0.0419
				hsa-miR-6716-3p	7.17	13.52	2.62	3.63	2.73	-0.29	0.0002	0.038
*RAC2*	22.11	37.39	0.59	hsa-miR-150-5p	14.9	20.24	39.17	38.83	0.38	0.39	<.0001	0.0203
				hsa-miR-203a	12.52	13.86	3.7	4.29	3.38	-0.28	<.0001	0.0203
				**hsa-miR-650**	4.51	5.86	16.6	9.34	0.27	0.36	<.0001	0.0203
*ROCK2*	447.77	282.59	1.58	**hsa-miR-1243**	1.48	1.96	3.2	1.82	0.46	-0.24	0.0006	0.0407
				**hsa-miR-17-5p**	61.04	48.49	16.38	10.13	3.73	0.32	<.0001	0.0116
				**hsa-miR-19b-3p**	29.8	23.72	10.42	9.70	2.86	0.26	0.0002	0.0163
				**hsa-miR-204-3p**	0.92	1.37	2.37	1.67	0.39	-0.28	<.0001	0.0116
				**hsa-miR-20a-5p**	70.78	59.44	17.61	12.25	4.02	0.3	<.0001	0.0116
				**hsa-miR-20b-5p**	17.65	15.14	3.3	3.59	5.35	0.33	<.0001	0.0116
				hsa-miR-21-5p	463.11	312.01	167.37	118.91	2.77	0.23	0.0009	0.0488
				hsa-miR-29a-3p	110.29	84.95	51.04	29.87	2.16	0.24	0.0007	0.0438
				hsa-miR-3651	58.66	34.62	25.92	12.63	2.26	0.24	0.0006	0.0407
				hsa-miR-4749-3p	8.01	4.10	12.04	3.97	0.67	–0.26	0.0002	0.0163
				hsa-miR-6515-5p	1.2	2.14	4.41	2.47	0.27	–0.23	0.0008	0.0465
				hsa-miR-663a	374.83	174.81	234.91	83.58	1.6	0.25	0.0002	0.0163
				hsa-miR-663b	65.5	24.80	32.21	14.68	2.03	0.34	<.0001	0.0116
				hsa-miR-92a-3p	121.6	104.09	41.18	24.92	2.95	0.3	<.0001	0.0116
				**hsa-miR-93-5p**	41.72	32.63	15.2	9.19	2.74	0.26	<.0001	0.0116
*MYC*	181.11	49	3.7	hsa-miR-1246	629.21	296.96	412.81	121.13	1.52	0.27	0.0002	0.0163
				hsa-miR-17-5p	61.04	48.49	16.38	10.13	3.73	0.35	<.0001	0.0136
				hsa-miR-19b-3p	29.8	23.72	10.42	9.70	2.86	0.27	0.0002	0.0163
				hsa-miR-20a-5p	70.78	59.44	17.61	12.25	4.02	0.33	<.0001	0.0136
				hsa-miR-20b-5p	17.65	15.14	3.3	3.59	5.35	0.31	0.0002	0.0163
				hsa-miR-3651	58.66	34.62	25.92	12.63	2.26	0.28	0.0003	0.0188
				hsa-miR-375	20.5	26.15	54.53	35.84	0.38	–0.29	<.0001	0.0136
				hsa-miR-501-3p	7.07	3.42	2.95	1.65	2.39	0.26	0.0003	0.0188
				hsa-miR-583	6.61	3.92	3.22	3.20	2.05	0.26	0.0004	0.0233
				hsa-miR-663a	374.83	174.81	234.91	83.58	1.6	0.28	0.0003	0.0188
				hsa-miR-663b	65.5	24.80	32.21	14.68	2.03	0.33	<.0001	0.0136
				hsa-miR-92a-3p	121.6	104.09	41.18	24.92	2.95	0.32	<.0001	0.0136
*PLCB2*	31.51	56.18	0.56	hsa-miR-150-5p	14.9	20.24	39.17	38.83	0.38	0.28	<.0001	0.0271
				hsa-miR-650	4.51	5.86	16.6	9.34	0.27	0.29	<.0001	0.0271
*DAAM2*	23.25	52.19	0.45	**hsa-miR-124-3p**	0.9	1.41	2.4	3.63	0.38	0.25	0.0006	0.0407
				**hsa-miR-133b**	1.71	4.98	6.94	8.13	0.25	0.28	<.0001	0.0203
				**hsa-miR-145-5p**	132.97	156.85	223.14	182.21	0.6	0.34	<.0001	0.0203
				**hsa-miR-150-5p**	14.9	20.24	39.17	38.83	0.38	0.24	0.0006	0.0407
				hsa-miR-195-5p	3.59	5.05	12.18	9.34	0.29	0.25	0.0004	0.0362
				hsa-miR-30a-5p	2.38	2.72	4.61	3.42	0.52	0.32	<.0001	0.0203
				hsa-miR-497-5p	1.77	3.05	7.12	5.05	0.25	0.25	0.0002	0.0271
*PRICKLE2*	30.26	50.46	0.6	**hsa-miR-133b**	1.71	4.98	6.94	8.13	0.25	0.25	0.0003	0.0488
				**hsa-miR-145-5p**	132.97	156.85	223.14	182.21	0.6	0.32	<.0001	0.0271
				**hsa-miR-193b-3p**	9.12	7.78	5.42	3.77	1.68	0.3	<.0001	0.0271
*PRKCB*	14.65	53.12	0.28	**hsa-miR-150-5p**	14.9	20.24	39.17	38.83	0.38	0.38	<.0001	0.0102
				**hsa-miR-203a**	12.52	13.86	3.7	4.29	3.38	–0.3	<.0001	0.0102
				hsa-miR-650	4.51	5.86	16.6	9.34	0.27	0.35	<.0001	0.0102
*CTNNB1*	581.34	358	1.62	**hsa-miR-21-3p**	22.68	12.22	9.89	5.94	2.29	0.24	0.0004	0.0407
				**hsa-miR-221-3p**	13.53	12.32	4.12	4.07	3.28	0.28	<.0001	0.0407
				**hsa-miR-27a-3p**	56.26	37.77	23.29	14.07	2.42	0.25	0.0003	0.0407
				hsa-miR-4315	0.21	0.93	2.62	1.27	0.08	–0.26	0.0002	0.0407

^1^Bold Text indicates seed match between mRNA and miRNA.

The majority of miRNA::mRNA associations were either both up-regulated or both down-regulated. Looking at seed-region matches between all dysregulated genes and all associated miRNAs there were 36 matches (Table 2 bold text indicates miRNA::mRNA seed matches). Of these 36 seed-region matches, seven had inverse associations between the miRNA and the mRNA. These included: *SFRP5* (FC 0.54) with miR-21-5p (FC 2.77), miR-3591-3p (FC 1.70), and miR-4251 (FC 1.86); *ROCK2* (FC 1.58) with miR-1243 (FC 0.46) and miR-204-3p (FC 0.39); *PRICKLE2* (FC 0.60) with miR-193b-3p (FC 1.68); and *PRKCB* (FC 0.28) with miR-203a (FC 3.38). However, the majority of seed matches were seen when the miRNA and mRNA were both up-regulated or were both down-regulated, suggesting a greater possibility of an indirect effect.

Evaluation of mRNA and miRNA with colorectal cancer-specific survival did not reveal any significant associations after adjustment for multiple comparisons.

## DISCUSSION

The Wnt-signaling pathway is often dysregulated in CRC [1, 13]. Genes within the pathway, including *APC*, are among the most commonly mutated genes in CRC [3, 13–15]. In our data, 43.5% of the genes identified in the KEGG Wnt-signaling pathway had significant up or down-regulated expression with a fold change of <0.67 or >1.50, which is slightly greater than when looking at all protein-coding genes in our RNA-Seq data. Thirteen of the 66 dysregulated genes in the Wnt-signaling pathway were associated with miRNA expression. There were 36 seed-region matches between the mRNAs and their associated miRNAs, suggesting that miRNAs had both direct and indirect effects on the Wnt-signaling pathway. Similar miRNAs were associated with genes in the same canonical/non-canonical pathway further suggesting that miRNA::mRNA associations were most likely from feedback and feedforward loops.

The Wnt-signaling pathway has three arms, the canonical Wnt/β-catenin pathway and two non-canonical Wnt-signaling pathways exist, Wnt/CA2+ and Wnt/planar cell polarity [4]. The Wnt/β-catenin pathway is most often studied with CRC and includes *CTNBB1* (that codes β-catenin), *APC*, *SMAD3* and *SMAD4*, c-*myc*, c-*jun*, *CCND1*, *TCF7L2*, *TP53*, *MMP7*, and *MAP3K7*, all genes that have been associated with CRC [1, 16–22]. β-catenin is a central component of this pathway in terms of regulation of cell growth and metastasis. Wnt-signaling triggers destabilization of β-catenin by the Axin complex. The tumor suppressor, *APC*, down-regulates β-catenin, as do Axins I and II (also tumor suppressors). Truncating mutations of *APC* alter the Axin binding sites for β-catenin and thus influences the stability of the β-catenin complex [23, 24]. Frizzled protein and Secreted frizzled-related protein (*SFRP*) modulate Wnt signaling through direct interaction with Wnt genes and have a role in regulating cell growth and differentiation. *SFRP5* has been shown to inhibit PAR1-induced β-catenin stabilization. Hypermethylation of *SFRP1* during chronic inflammation has been shown to lead to the occurrence of CRC [25]; *SFRP2* also is known to modulate Wnt signaling and can increase B-catenin expression [26].

In our study, expression of several Wnt genes, including *WNT5B*, *WNT1*, *WNT10B*, *WNT2B*, *WNT9A*, *WNT4*, *WNT10A*, *WNT16*, and *WNT8B* were down-regulated with FC of <0.67, while others such as *WNT2*, *WNT11*, and *WNT5A* were up-regulated. Also down-regulated at that level were *SFRP1* and *SFRP5*. *APC2* and *APC* were both statistically significantly down-regulated but with higher FCs (FCs 0.68 and 0.76 respectively). While *APC* was down-regulated in tumor tissue, this does not necessarily equate mutation, although roughly 80% of the tumors in this study had an *APC* mutation. *APC* mutations are usually stop mutations and frame shifts, which would lead to loss of functional protein and possibly less stable mRNA through nonsense-mediated RNA decay [27, 28]. *AXIN2*, was highly up-regulated in our data. Additionally, expression of *CTNNB1* was increased in tumor samples in our data, suggesting that several elements of Wnt-signaling were dysregulated and that the stabilization of β-catenin needed to control cell growth was destroyed. Down-stream of *CTNNB1* in this arm of the Wnt-signaling pathway, *LEF1*, *MYC*, *CCND1*, and *MMP7* were up-regulated, and *TCF7L1* down-regulated.

Several miRNAs were associated with differential gene expression in the canonical Wnt/β-catenin pathway in our data. Most notably, *SFRP4* and *SFRP5* had nine and 18 associations with miRNAs, *CCND1* was associated with 11 miRNAs, *MYC* was associated with 12 miRNA, and *CTNNB1* was associated with four miRNAs. *SFRP4* and *SFRP5* were both associated with let-7i-5p and with 193b-3p. MiRNA-201a had a seed match with both *PRKCB* and *CCND1*. MiR-21-3p had a seed-region match with *CTNNB1* while miR-21-5p had a seed-region match with *SFRP5* and also was associated with *CCND1*. There were several miRNAs that had seed region matches with *CCND1* that also were associated with *MYC*, including miR-17-5p, miR-19b-3p, miR-20a-5p and miR-20b-5p. Seed region matches suggest a greater likelihood of a direct association between the mRNA and the miRNA. In most instances of seed region matches in our data both the miRNA and mRNA were either up-regulated or down-regulated, suggesting feedback or feedforward loops influenced the expression profiles though an indirect mechanism. Our data suggest that within the Wnt-signaling pathway miRNAs are associated both directly and indirectly with TG to alter gene expression.

The non-canonical arms of the Wnt-signaling pathways are thought to have indirect associations to the Wnt/β-catenin pathway [1, 2, 7]. Both the Wnt/CA2+ and Wnt/PCP pathways have genes that were either up or down-regulated in our data and some of these genes were associated with miRNA differential expression. The Wnt/PCP pathway is associated with cell orientation during development but is thought also to have a role in metastasis [29]. The Wnt/CA2+-signaling pathway controls intracellular calcium influence and can activate several downstream kinases including *CAMKII* which has been shown to inhibit β-catenin-dependent transcription [2]. *WNT11* is central to the WNT/PCP pathway while *WNT5A* is involved in the Wnt/CA2+ pathway. Both *WNT11* and *WNT5A* were significantly up-regulated in our data.

Several genes in the Wnt/PCP pathway were differentially expressed in our CRC samples. Several of these genes, including *PRICKLE2*, *DAAM2*, *ROCK2*, and *RAC2*, also were associated with miRNA expression. *RAC2* had a seed-region match with miR-650, *ROCK2* had a seed-region match with seven miRNAs, with miR-1243 and miR-204-3p expression being inversely related to *ROCK2* expression; *DAAM2* had seed region matches with four miRNAs and *PRICKLE2* has a seed region match with three miRNAs. There was overlap between miRNAs differentially expressed and genes within the pathways, such as miR-133b being associated with both *DAAM2* and *PRICKLE2*. Again, these findings would support that miRNAs have both direct and indirect associations with these pathways.

The Wnt-signaling pathway has been associated with miRNAs in other studies. Several of these studies have been reviewed by Peng and colleagues [12]. While the review by Peng included non-CRC associations, there were several genes in the Wnt-signaling pathway that were associated with miRNAs and CRC. They reported CRC-specific previous associations between miR-101 and miR-320 and β-catenin, miR-224 and *GSK3β*, and miR-490-3p and *FRAT1*. We did not see any of these associations, however we only examined miRNA associations with genes that had a more meaningful FC and since we were examining multiple genes and miRNAs, our correction for multiple comparisons was greater. MiR-221 has been shown to be associated with β-catenin pathways and *MYC* [30]. In our data miR-221-3p was associated with *CTNNB1* and *CCND1* (both with seed matches). MiR-29 and miR-30e have been shown to influence Wnt signaling; miR-23b has been shown to inhibit *FZD7* translation; miR93 has been shown to down-regulate expression of genes encoding β-catenin, Axin, Myc and Cyclin D; and miR-135a/5 have been shown to suppress *APC* expression [4]. In our data miR-29b-3p and miR-93-5p had a seed region match with *CCND1*, and miR-29a-3p was also associated with *SFRP5* and *ROCK2*; while miR-30a-5p was associated with *DAAM2*, suggesting miRNA regulation of both the canonical and non-canonical Wnt-signaling pathways.

There are strengths and limitations of this study to consider. Although our sample size is small, it is one of the largest available samples with paired tumor/normal data. We focused only those genes that were statistically significant and also had a FC of 1.5 or greater or 0.67 or less. Using these criteria, we did not examine all statistically significant genes and miRNAs that were differentially expressed. Thus, genes like *APC*, which were statistically significantly down-regulated but the FC was greater than 0.67, were not examined further with miRNAs. A biologically important FC is not well defined, and by using set values for further follow-up we could have missed Wnt-signaling genes associated with miRNAs. Additionally, in our current analysis, we utilized a negative binomial model with a random subject effect. Previously we reported results from DESEQ2 for some of these genes; DESEQ2 uses a fixed effect model and additional normalization and variance reduction methods. Our results vary slightly between the two analytic methods in terms of FC and adjusted *p* values, although the interpretation of findings is consistent. We exclusively used the KEGG pathway database to identify Wnt-signaling pathway genes. Some genes, such as *WTX* and *AMER1*, that have been shown to influence Wnt-signaling [31, 32] were not considered by KEGG to be part of the Wnt-signaling pathway, and therefore we did not include them in our analysis. Thus, other genes may importantly alter Wnt-signaling as well as influence miRNA expression and subsequently mRNA expression within those components of Wnt-signaling that we examined. When evaluating miRNA with mRNAs, we could miss important gene associations since miRNAs have their impact post-transcriptionally. However, much of the current information on miRNA target genes comes from gene expression data and association observed may have important biological meaning, but must be acknowledged as being incomplete [33, 34].

Given the number of genes in the Wnt-signaling pathway that were dysregulated in our study, our data support the importance of this pathway in CRC. Our data also support the hypothesis that miRNAs are involved in this signaling pathway, either through direct binding to the mRNA or through indirect mechanisms. We encourage others to both replicate these findings and to conduct targeted research on the identified associations to further our understanding of this important signaling pathway in the carcinogenic process.

## METHODS

### Study participants

Study participants come from two population-based case-control studies that included all incident colon and rectal cancer patients diagnosed between 30 to 79 years of age in Utah or who were members of the Kaiser Permanente of Northern California (KPNC). Participants were non-Hispanic white, Hispanic, or black for the colon cancer study; the rectal cancer study also included people of Asian race [35, 36]. Case diagnosis was verified by tumor registry data as a first primary adenocarcinoma of the colon or rectum and occurred between October 1991 and September 1994 (colon study) and between May 1997 and May 2001 (rectal study) [37]. The Institutional Review Boards at the University of Utah and at KPNC approved the study.

### RNA processing

Formalin-fixed paraffin embedded tissue from the initial biopsy or surgery was used to extract RNA. RNA was extracted, isolated and purified from carcinoma tissue and adjacent normal mucosa as previously described [38]. We observed no differences in RNA quality based on age of the tissue.

### mRNA: RNA-seq sequencing library preparation and data processing

Total RNA from 245 colorectal carcinoma and normal mucosa pairs was chosen for sequencing based on availability of RNA and high quality miRNA data in order to have both mRNA and miRNA from the same individuals; the 217 pairs that passed quality control (QC) were used in these analyses [39]. RNA library construction was performed with the Illumina TruSeq Stranded Total RNA Sample Preparation Kit with Ribo-Zero. The samples were then fragmented and primed for cDNA synthesis, adapters were then ligated onto the cDNA, and the resulting samples were then amplified using PCR; the amplified library was then purified using Agencount AMPure XP beads. A more detailed description of the methods can be found in our previous work [40]. Illumina TruSeq v3 single read flow cell and a 50 cycle single-read sequence run were performed on an Illumina HiSeq instrument. Reads were aligned to a sequence database containing the human genome (build GRCh37/hg19, February 2009 from genome.ucsc.edu) and alignment was performed using novoalign v2.08.01. Total gene counts were calculated for each exon and UTR of the genes using gene coordinates obtained from http://genome.ucsc.edu. We disregarded genes that were not expressed in our RNA-Seq data or for which the expression was missing for the majority of samples [40].

### miRNA

The Agilent Human miRNA Microarray V19.0 was used. Data were required to pass stringent QC parameters established by Agilent that included tests for excessive background fluorescence, excessive variation among probe sequence replicates on the array, and measures of the total gene signal on the array to assess low signal. Samples failing to meet quality standards were re-labeled, hybridized to arrays, and re-scanned. If a sample failed QC assessment a second time, the sample was excluded from analysis. The repeatability associated with this microarray was extremely high (*r* = 0.98) [37]; comparison of miRNA expression levels obtained from the Agilent microarray to those obtained from qPCR had an agreement of 100% in terms of directionality of findings and the FCs were almost identical [41]. To normalize differences in miRNA expression that could be attributed to the array, amount of RNA, location on array, or factors that could erroneously influence miRNA expression levels, total gene signal was normalized by multiplying each sample by a scaling factor which was the median of the 75^th^ percentiles of all the samples divided by the individual 75^th^ percentile of each sample [42].

### *WNT*-signaling genes

The Kyoto Encyclopedia of Genes and Genomes (KEGG) (www.genome.jp/kegg-gin/show_pathway?hsa04310) Pathway map for Wnt-signaling was used to identify genes associated with the canonical and non-canonical Wnt-signaling pathway. Using this map, we identified 138 genes (Supplementary Table 2) in this signaling pathway.

### Statistical methods

We utilized negative binomial mixed effects model in SAS (accounting for carcinoma/normal status as well as subject effect) to determine which genes in the Wnt-signaling pathway had a significant difference in expression between individually paired colorectal carcinoma and normal mucosa and their related fold changes (FC). In the negative binomial model we offset the overall exposure as the log of the expression of all identified protein-coding genes (*n* = 17461). The Benjamini and Hochberg [43] procedure was used to control the false discovery rate (FDR) using a value of 0.05 or less. A FC greater than one indicates a positive differential expression (i.e. up-regulated in carcinoma tissue) while a FC between zero and one indicates a negative differential expression (i.e. down-regulated in carcinoma tissue). We generated the level of expression of each gene by dividing the total expression for that gene in an individual by the total expression of all protein-coding genes per million transcripts (RPMPCG or reads per million protein-coding genes). We considered overall CRC differential expression as well as differential expression for microsatellite unstable (MSI) and stable (MSS) tumors separately since the Wnt-signaling pathway is thought to have a larger role in MSS tumor development.

We arbitrarily focused on those genes and miRNAs with FCs of ≥1.50 or ≤0.67 in order to have more meaningful differences between tumor and normal samples. There were 814 miRNAs expressed in greater than 20% of normal colorectal mucosa samples that were analyzed; differential expression was calculated using subject-level paired data as the expression in the carcinoma tissue minus the expression in the normal mucosa. In these analyses, we fit a least squares linear regression model to the RPMPCG differential expression levels and miRNA differential expression levels. *P*-values were generated using the bootstrap method by creating a distribution of 10,000 F statistics derived by resampling the residuals from the null hypothesis model of no association between gene expression and miRNA expression using the boot package in R. Linear models were adjusted for age and sex. Multiplicity adjustments for gene/miRNA associations were made at the gene level using the FDR by Benjamini and Hochberg [43].

### Bioinformatics analysis

We analyzed miRNAs and targeted mRNAs for seed region matches. The mRNA 3’ UTR FASTA as well as the seed region sequence of the associated miRNA were analyzed to determine seed region pairings between miRNA and mRNA. MiRNA seed regions were calculated as described in our previous work [44]; we calculated and included seeds of six, seven, and eight nucleotides in length. Our hypothesis is that a seed match would increase the likelihood that identified genes associated with a specific miRNA were more likely to have a direct association given a higher propensity for binding. As miRTarBase [33] uses findings from many different investigations spanning across years and alignments, we used FASTA sequences generated from both GRCh37 and GRCh38 Homo sapiens alignments, using UCSC Table Browser (https://genome.ucsc.edu/cgi-bin/hgTables) [45]. We downloaded FASTA sequences that matched our Ensembl IDs and had a consensus coding sequences (CCDS) available. Analysis was done using scripts in R 3.2.3 and in perl 5.018002.

## SUPPLEMENTARY MATERIALS AND TABLES








