# Supplementary material for: Expression of Wnt-signaling pathway genes and their associations with miRNAs in colorectal cancer
Source: Oncotarget. 2017 Dec 23;9(5):6075–85. doi: 10.18632/oncotarget.23636 (PMC5814196; doi:10.18632/oncotarget.23636)
Supplement: Supplementary file 1 [file oncotarget-09-6075-s001.pdf]

Expression of Wnt-signaling pathway genes and their associations with miRNAs in colorectal cancer

SUPPLEMENTARY MATERIALS

Supplementary Table 1: Associations between KEGG Wnt-signaling pathway genes and differential expression in CRC tumor and normal mucosa. See Supplementary_Table_1

Supplementary Table 2: Summary of Wnt-signaling pathway genes. See Supplementary_Table_2

Supplementary Table 3: Wnt-signaling pathway genes with MSS tumors only. See Supplementary_Table_3
