# Supplementary material for: Expression of Wnt-signaling pathway genes and their associations with miRNAs in colorectal cancer
Source: Oncotarget. 2017 Dec 23;9(5):6075–85. doi: 10.18632/oncotarget.23636 (PMC5814196; doi:10.18632/oncotarget.23636)
Supplement: Supplementary file 2 [file oncotarget-09-6075-s002.docx]

| Supplementary Table 1: Associations between KEGG Wnt-signaling pathway genes and differential expression in CRC tumor and normal mucosa | | | | | | |  |  |
| --- | --- | --- | --- | --- | --- | --- | --- | --- |
| Gene Name | Tumor Mean | Tumor SD | Normal Mean | Normal SD | Fold Change | P-Value | | Adjusted P-Value |
| *SFRP1* | 2.28 | 1.78 | 36.37 | 2.65 | 0.06 | 4.03E-53 | | 1.11E-51 |
| *PPP3R2* | 0.2 | 13.66 | 0.93 | 13.37 | 0.21 | 3.56E-07 | | 6.55E-07 |
| *CAMK2A* | 6.11 | 58.48 | 22.22 | 61.42 | 0.27 | 8.35E-35 | | 7.69E-34 |
| *PRKCB* | 14.65 | 27.12 | 53.12 | 27.10 | 0.28 | 1.52E-41 | | 2.33E-40 |
| *WNT5B* | 5.61 | 28.78 | 19.25 | 21.35 | 0.29 | 2.69E-37 | | 2.85E-36 |
| *WNT1* | 0.34 | 2.58 | 1.09 | 5.61 | 0.32 | 2.17E-07 | | 4.10E-07 |
| *MAPK10* | 9.26 | 75.79 | 28.1 | 57.62 | 0.33 | 8.93E-34 | | 7.25E-33 |
| *WNT10B* | 1.29 | 55.13 | 3.57 | 46.65 | 0.36 | 1.55E-13 | | 3.97E-13 |
| *PRKACB* | 62.6 | 24.34 | 162.73 | 12.93 | 0.38 | 5.72E-40 | | 7.90E-39 |
| *WNT2B* | 21.06 | 6.49 | 54.04 | 20.97 | 0.39 | 8.64E-49 | | 1.70E-47 |
| *WNT9A* | 2.48 | 17.24 | 5.89 | 20.14 | 0.42 | 1.40E-14 | | 3.78E-14 |
| *CAMK2B* | 1.17 | 46.18 | 2.63 | 46.58 | 0.44 | 2.48E-06 | | 4.28E-06 |
| *DAAM2* | 23.25 | 24.10 | 52.19 | 22.45 | 0.45 | 1.11E-38 | | 1.39E-37 |
| *TBL1Y* | 0.24 | 2.43 | 0.51 | 3.71 | 0.47 | 6.71E-03 | | 9.35E-03 |
| *TCF7L1* | 8.1 | 32.86 | 16.73 | 17.76 | 0.48 | 2.92E-21 | | 1.22E-20 |
| *NFATC1* | 12.01 | 26.60 | 22.97 | 35.39 | 0.52 | 3.23E-17 | | 1.01E-16 |
| *SFRP5* | 0.67 | 38.66 | 1.23 | 40.21 | 0.54 | 1.89E-04 | | 2.93E-04 |
| *WNT4* | 4.08 | 48.44 | 7.34 | 41.15 | 0.56 | 3.96E-11 | | 9.10E-11 |
| *PRKACG* | 0.17 | 29.58 | 0.31 | 5.45 | 0.56 | 1.84E-03 | | 2.62E-03 |
| *PLCB2* | 31.51 | 18.77 | 56.18 | 19.14 | 0.56 | 4.27E-19 | | 1.55E-18 |
| *RAC2* | 22.11 | 0.98 | 37.39 | 1.75 | 0.59 | 4.70E-16 | | 1.32E-15 |
| *WNT10A* | 4.4 | 11.80 | 7.39 | 6.71 | 0.6 | 1.08E-05 | | 1.73E-05 |
| *PRICKLE2* | 30.26 | 10.36 | 50.46 | 12.24 | 0.6 | 1.48E-18 | | 5.09E-18 |
| *WNT7A* | 0.35 | 58.82 | 0.55 | 68.75 | 0.64 | 8.33E-02 | | 9.83E-02 |
| *CAMK2D* | 90.24 | 23.16 | 139.09 | 24.83 | 0.65 | 2.48E-28 | | 1.56E-27 |
| *WNT16* | 0.81 | 37.77 | 1.23 | 36.58 | 0.66 | 1.66E-02 | | 2.22E-02 |
| *WNT8B* | 1.75 | 16.87 | 2.64 | 18.76 | 0.66 | 5.08E-04 | | 7.54E-04 |
| *SOX17* | 1.28 | 37.23 | 1.93 | 51.70 | 0.67 | 1.52E-03 | | 2.19E-03 |
| *APC2* | 20.64 | 51.71 | 30.36 | 34.89 | 0.68 | 6.95E-13 | | 1.71E-12 |
| *PRICKLE1* | 7.03 | 284.07 | 10.13 | 130.45 | 0.69 | 1.45E-05 | | 2.30E-05 |
| *TCF7L2* | 128.93 | 28.05 | 183 | 20.25 | 0.7 | 3.26E-21 | | 1.33E-20 |
| *NFATC2* | 60.6 | 7.51 | 85.42 | 6.82 | 0.71 | 3.55E-09 | | 7.78E-09 |
| *WNT9B* | 0.68 | 40.27 | 0.95 | 34.00 | 0.72 | 8.03E-02 | | 9.55E-02 |
| *PSEN1* | 85.05 | 34.66 | 113.6 | 16.91 | 0.75 | 8.89E-21 | | 3.50E-20 |
| *SMAD4* | 89.84 | 3.98 | 119.48 | 47.10 | 0.75 | 7.97E-17 | | 2.39E-16 |
| *APC* | 101.79 | 3.99 | 133.72 | 1.66 | 0.76 | 5.48E-19 | | 1.94E-18 |
| *FZD5* | 227.88 | 13.94 | 296.66 | 2.34 | 0.77 | 1.06E-13 | | 2.76E-13 |
| *SFRP2* | 26.18 | 46.21 | 33.76 | 8.10 | 0.78 | 3.28E-02 | | 4.15E-02 |
| *FRAT1* | 6.25 | 35.14 | 8.01 | 29.37 | 0.78 | 1.34E-03 | | 1.95E-03 |
| *PPP3CC* | 17.08 | 27.67 | 20.97 | 27.89 | 0.81 | 4.27E-04 | | 6.48E-04 |
| *PPARD* | 59.29 | 24.67 | 72.71 | 26.41 | 0.82 | 1.26E-07 | | 2.41E-07 |
| *CCND3* | 33.57 | 2.54 | 40.72 | 1.79 | 0.82 | 1.67E-06 | | 2.92E-06 |
| *PRKCA* | 69.4 | 2.92 | 83.99 | 1.41 | 0.83 | 8.91E-08 | | 1.76E-07 |
| *CAMK2G* | 72.94 | 7.50 | 86.91 | 21.98 | 0.84 | 1.49E-08 | | 3.12E-08 |
| *WNT6* | 0.63 | 143.29 | 0.75 | 57.86 | 0.84 | 4.14E-01 | | 4.57E-01 |
| *SMAD3* | 106.05 | 5.39 | 124.63 | 16.27 | 0.85 | 1.09E-06 | | 1.96E-06 |
| *EP300* | 293.9 | 13.39 | 339.29 | 3.50 | 0.87 | 2.31E-14 | | 6.12E-14 |
| *PRKACA* | 53.45 | 26.10 | 60.68 | 32.35 | 0.88 | 3.93E-06 | | 6.62E-06 |
| *RBX1* | 20.82 | 14.70 | 23.31 | 17.98 | 0.89 | 9.83E-03 | | 1.34E-02 |
| *CTBP1* | 166.7 | 31.88 | 185.94 | 29.01 | 0.9 | 3.41E-06 | | 5.80E-06 |
| *FZD1* | 25.04 | 88.15 | 27.58 | 77.61 | 0.91 | 5.98E-02 | | 7.36E-02 |
| *NFATC4* | 27.58 | 43.40 | 30.31 | 24.38 | 0.91 | 5.60E-02 | | 6.96E-02 |
| *DAAM1* | 52.46 | 11.87 | 56.81 | 17.32 | 0.92 | 4.89E-02 | | 6.13E-02 |
| *SERPINF1* | 29.68 | 1.57 | 31.94 | 2.03 | 0.93 | 1.91E-01 | | 2.18E-01 |
| *SIAH1* | 44.02 | 18.95 | 46.13 | 11.68 | 0.95 | 1.63E-01 | | 1.88E-01 |
| *PPP3CB* | 55.64 | 480.51 | 58.21 | 279.16 | 0.96 | 2.28E-01 | | 2.56E-01 |
| *CREBBP* | 264.23 | 21.68 | 273.44 | 23.21 | 0.97 | 6.13E-02 | | 7.42E-02 |
| *WNT3A* | 0.35 | 1.49 | 0.36 | 2.75 | 0.97 | 8.90E-01 | | 9.03E-01 |
| *INVS* | 51.74 | 10.12 | 52.96 | 12.63 | 0.98 | 5.61E-01 | | 6.10E-01 |
| *TCF7* | 94.88 | 0.93 | 95.43 | 2.75 | 0.99 | 8.82E-01 | | 9.01E-01 |
| *CTBP2* | 154.29 | 6.99 | 153.97 | 4.56 | 1 | 9.43E-01 | | 9.43E-01 |
| *FRAT2* | 22.49 | 14.76 | 22.38 | 24.62 | 1.01 | 9.06E-01 | | 9.13E-01 |
| *NFATC3* | 159.9 | 10.81 | 158.77 | 19.58 | 1.01 | 7.71E-01 | | 8.00E-01 |
| *BTRC* | 40.53 | 18.58 | 40.22 | 19.56 | 1.01 | 8.44E-01 | | 8.70E-01 |
| *NLK* | 48.11 | 10.89 | 47.34 | 27.14 | 1.02 | 6.50E-01 | | 6.90E-01 |
| *JUN* | 214.48 | 174.03 | 211.03 | 109.55 | 1.02 | 7.02E-01 | | 7.40E-01 |
| *MAPK8* | 62.01 | 34.69 | 60.98 | 45.63 | 1.02 | 6.19E-01 | | 6.62E-01 |
| *PPP3CA* | 81.01 | 26.87 | 79.4 | 23.89 | 1.02 | 6.04E-01 | | 6.51E-01 |
| *MAPK9* | 65.92 | 6.04 | 64.12 | 8.88 | 1.03 | 4.12E-01 | | 4.57E-01 |
| *VANGL2* | 33.48 | 59.45 | 31.98 | 43.31 | 1.05 | 5.54E-01 | | 6.07E-01 |
| *DVL3* | 147.55 | 127.15 | 140.88 | 35.62 | 1.05 | 3.10E-02 | | 3.96E-02 |
| *FZD4* | 43.46 | 37.18 | 41.32 | 3.82 | 1.05 | 2.19E-01 | | 2.48E-01 |
| *CHD8* | 150.51 | 20.34 | 142.32 | 34.33 | 1.06 | 1.09E-02 | | 1.48E-02 |
| *PLCB3* | 98.35 | 31.13 | 92.06 | 11.54 | 1.07 | 6.04E-02 | | 7.38E-02 |
| *DVL2* | 29.71 | 36.20 | 27.7 | 35.25 | 1.07 | 1.03E-01 | | 1.20E-01 |
| *FZD9* | 0.36 | 6.57 | 0.33 | 9.21 | 1.08 | 7.28E-01 | | 7.61E-01 |
| *CSNK2B* | 78.25 | 198.60 | 70.03 | 22.39 | 1.12 | 2.92E-04 | | 4.48E-04 |
| *FBXW11* | 71.07 | 52.93 | 63.57 | 31.11 | 1.12 | 9.37E-04 | | 1.38E-03 |
| *SKP1* | 91.51 | 36.34 | 81.23 | 48.23 | 1.13 | 7.71E-05 | | 1.21E-04 |
| *FZD8* | 14.6 | 43.67 | 12.74 | 106.70 | 1.15 | 6.65E-02 | | 7.99E-02 |
| *CSNK1A1* | 295.14 | 3.22 | 257.01 | 6.99 | 1.15 | 1.92E-08 | | 3.95E-08 |
| *MAP3K7* | 72.49 | 37.20 | 62.09 | 56.31 | 1.17 | 9.69E-07 | | 1.76E-06 |
| *SENP2* | 59.67 | 38.90 | 50.31 | 47.04 | 1.19 | 1.14E-06 | | 2.02E-06 |
| *LRP5* | 189.19 | 37.23 | 159.09 | 5.87 | 1.19 | 8.02E-09 | | 1.73E-08 |
| *VANGL1* | 63.68 | 12.35 | 52.98 | 27.03 | 1.2 | 9.46E-06 | | 1.54E-05 |
| *LRP6* | 163.84 | 25.29 | 136.27 | 30.52 | 1.2 | 2.90E-11 | | 6.78E-11 |
| *GSK3B* | 169.34 | 53.39 | 139.86 | 75.28 | 1.21 | 9.49E-15 | | 2.62E-14 |
| *PORCN* | 9.07 | 43.88 | 7.49 | 41.82 | 1.21 | 1.68E-02 | | 2.23E-02 |
| *AXIN1* | 96.49 | 6.20 | 79.64 | 12.60 | 1.21 | 2.39E-08 | | 4.84E-08 |
| *DVL1* | 71.99 | 27.01 | 58.73 | 32.27 | 1.23 | 1.42E-08 | | 3.01E-08 |
| *CSNK1E* | 170.22 | 0.94 | 138.02 | 1.09 | 1.23 | 1.45E-17 | | 4.76E-17 |
| *CXXC4* | 12.06 | 1.28 | 9.67 | 1.97 | 1.25 | 1.81E-02 | | 2.36E-02 |
| *TBL1XR1* | 383.96 | 8.80 | 303.09 | 3.17 | 1.27 | 5.61E-18 | | 1.89E-17 |
| *CTNNBIP1* | 27.92 | 13.20 | 21.41 | 9.93 | 1.3 | 2.42E-07 | | 4.51E-07 |
| *FZD2* | 1.95 | 9.68 | 1.49 | 2.23 | 1.31 | 2.24E-02 | | 2.89E-02 |
| *FZD7* | 18.1 | 14.32 | 13.41 | 15.63 | 1.35 | 7.50E-06 | | 1.23E-05 |
| *RHOA* | 282.98 | 1.63 | 209.5 | 2.63 | 1.35 | 5.89E-27 | | 3.54E-26 |
| *RAC3* | 2.78 | 43.11 | 2.05 | 48.98 | 1.36 | 9.11E-03 | | 1.26E-02 |
| *CUL1* | 74.71 | 36.74 | 53.22 | 36.12 | 1.4 | 1.56E-19 | | 5.83E-19 |
| *RAC1* | 180.81 | 67.38 | 127.6 | 57.64 | 1.42 | 3.51E-26 | | 2.02E-25 |
| *TBL1X* | 49.2 | 3.60 | 34.04 | 6.41 | 1.45 | 3.39E-10 | | 7.54E-10 |
| *PPP3R1* | 89.41 | 30.44 | 61.65 | 27.41 | 1.45 | 1.55E-23 | | 6.68E-23 |
| *DKK1* | 0.75 | 84.12 | 0.51 | 106.92 | 1.47 | 1.54E-01 | | 1.79E-01 |
| *PRKCG* | 3.38 | 17.87 | 2.29 | 28.27 | 1.48 | 1.74E-02 | | 2.29E-02 |
| *FZD6* | 34.42 | 21.66 | 23.28 | 19.12 | 1.48 | 1.61E-13 | | 4.05E-13 |
| *CSNK2A1* | 138.89 | 1.85 | 92.92 | 2.88 | 1.49 | 5.00E-25 | | 2.55E-24 |
| *ROCK2* | 447.77 | 17.67 | 282.59 | 12.85 | 1.58 | 1.95E-32 | | 1.49E-31 |
| *CCND2* | 773.45 | 0.53 | 483.06 | 0.97 | 1.6 | 3.23E-16 | | 9.30E-16 |
| *CTNNB1* | 581.34 | 5.48 | 358 | 7.41 | 1.62 | 8.86E-44 | | 1.53E-42 |
| *CACYBP* | 36.69 | 16.29 | 20.83 | 16.87 | 1.76 | 4.51E-26 | | 2.49E-25 |
| *TP53* | 105.07 | 13.11 | 59.63 | 44.31 | 1.76 | 3.25E-24 | | 1.45E-23 |
| *BAMBI* | 9.61 | 38.22 | 5.2 | 44.22 | 1.85 | 1.14E-07 | | 2.22E-07 |
| *WNT5A* | 61.7 | 170.94 | 33.36 | 105.37 | 1.85 | 1.86E-16 | | 5.46E-16 |
| *GPC4* | 54.88 | 248.94 | 28.91 | 55.60 | 1.9 | 4.30E-25 | | 2.28E-24 |
| *RUVBL1* | 45.79 | 12.67 | 24.09 | 10.06 | 1.9 | 2.34E-36 | | 2.31E-35 |
| *PLCB1* | 46.07 | 3.71 | 23.57 | 3.01 | 1.95 | 1.69E-17 | | 5.41E-17 |
| *PLCB4* | 325.61 | 2.06 | 166.3 | 4.90 | 1.96 | 7.44E-17 | | 2.28E-16 |
| *CSNK2A2* | 46.72 | 27.49 | 23.14 | 23.30 | 2.02 | 1.14E-30 | | 7.89E-30 |
| *WNT7B* | 1.05 | 19.37 | 0.5 | 18.77 | 2.08 | 4.67E-04 | | 7.01E-04 |
| *FZD3* | 46.01 | 47.42 | 21.78 | 48.69 | 2.11 | 1.64E-24 | | 7.79E-24 |
| *CSNK2A1P* | 1.98 | 18.89 | 0.91 | 6.72 | 2.17 | 1.29E-12 | | 3.12E-12 |
| *DKK4* | 0.92 | 20.07 | 0.38 | 11.59 | 2.41 | 4.49E-03 | | 6.33E-03 |
| *CCND1* | 317.79 | 12.22 | 122.64 | 10.48 | 2.59 | 1.53E-59 | | 1.05E-57 |
| *WNT3* | 1.84 | 153.52 | 0.64 | 122.01 | 2.86 | 3.38E-10 | | 7.54E-10 |
| *LEF1* | 41.11 | 120.58 | 13.71 | 119.88 | 3 | 2.03E-38 | | 2.33E-37 |
| *FZD10* | 4.67 | 14.09 | 1.42 | 11.41 | 3.28 | 4.05E-08 | | 8.09E-08 |
| *FOSL1* | 14.16 | 2.28 | 4.14 | 1.92 | 3.42 | 6.40E-20 | | 2.45E-19 |
| *MYC* | 181.11 | 11.65 | 49 | 11.75 | 3.7 | 5.76E-55 | | 1.99E-53 |
| *DKK2* | 5.17 | 36.81 | 1.33 | 18.48 | 3.88 | 3.60E-12 | | 8.57E-12 |
| *WIF1* | 2.25 | 37.12 | 0.57 | 2.44 | 3.94 | 4.15E-06 | | 6.90E-06 |
| *AXIN2* | 329.32 | 2.70 | 75.96 | 1.49 | 4.34 | 5.36E-60 | | 7.40E-58 |
| *WNT11* | 16.64 | 0.84 | 3.25 | 3.46 | 5.12 | 3.26E-24 | | 1.45E-23 |
| *NKD2* | 34.97 | 1.29 | 5.01 | 1.19 | 6.97 | 4.82E-52 | | 1.11E-50 |
| *SFRP4* | 35.37 | 16.71 | 5.03 | 18.06 | 7.03 | 1.57E-34 | | 1.36E-33 |
| *NKD1* | 159.58 | 24.01 | 18.19 | 22.47 | 8.77 | 1.78E-56 | | 8.19E-55 |
| *WNT2* | 11.03 | 52.37 | 1.11 | 43.66 | 9.9 | 5.05E-32 | | 3.67E-31 |
| *MMP7* | 16.5 | 34.62 | 1.27 | 24.97 | 12.97 | 1.47E-24 | | 7.24E-24 |
| *NOTUM* | 14.29 | 4.67 | 0.74 | 2.27 | 19.34 | 3.47E-29 | | 2.28E-28 |
