# Supplementary material for: Expression of Wnt-signaling pathway genes and their associations with miRNAs in colorectal cancer
Source: Oncotarget. 2017 Dec 23;9(5):6075–85. doi: 10.18632/oncotarget.23636 (PMC5814196; doi:10.18632/oncotarget.23636)
Supplement: Supplementary file 3 [file oncotarget-09-6075-s003.docx]

| Supplemental Table 2: Summary of Wnt-Signaling Pathway Genes | |  |
| --- | --- | --- |
| Gene | Name | Alias |
| *APC* | adenomatous polyposis coli |  |
| *APC2* | adenomatous polyposis coli 2 |  |
| *AXIN1* | axin 1 |  |
| *AXIN2* | axin2 |  |
| *BAMBI* | BMP and activin membrane bound inhibitor |  |
| *BTRC* | beta-transducin repeat containing E3 ubiquitin protein ligase | FBXW1, B-TrCP |
| *CACYBP* | calcyclin binding protein | SIP, GIG5 |
| *CAMK2A* | calcium/calmodulin dependent protein kinase II alpha | CAMKA |
| *CAMK2B* | calcium/calmodulin dependent protein kinase II beta | CAM2, CAMKB, CAMKII |
| *CAMK2D* | calcium/calmodulin dependent protein kinase II delta | CAMKD |
| *CAMK2G* | calcium/calmodulin dependent protein kinase II gamma | CAMK, CAMKG |
| *CCND1* | cyclin D1 |  |
| *CCND2* | cyclin D2 |  |
| *CCND3* | cyclin D3 |  |
| *CER1* | cerberus 1, DAN family BMP antagonist | DNAD4 |
| *CHD8* | chromodomain helicase DNA binding protein 8 | AUTS18; HELSFN1 |
| *CREBBP* | CREB binding protein |  |
| *CSNK1A1* | Casein Kinase 1 alpha 1 | CK1, CK!a |
| *CSNK1A1L* | Casein Kinase 1 alpha 1 Like | CK1, CK!a |
| *CSNK1E* | Casein Kinase 1 epsilon | HCKIE |
| *CSNK2A1* | Casein kinase 2 alpha 1 | CKII; CK1A1 |
| *CSNK2A2* | Casein kinase 2 alpha 2 | CK2A2; CSNK2A1 |
| *CSNK2A3* | Casein kinase 2 alpha 3 | CSNK2A1P |
| *CSNK2B* | Casein kinase 2 beta | G5A; CK2B; CK2N; CSK2B |
| *CTBP1* | C-Terminal Binding Protein 1 |  |
| *CTBP2* | C-Terminal Binding Protein 2 |  |
| *CTNNB1* | Catenin (Cadherin-Associated Protein), Beta | β-catenin |
| *CTNNBIP1* | Beta -catenin-interacting protein 1 | Tcf-4 |
| *CUL1* | Cullin 1 |  |
| *CXXC4* | CXXC-type zinc finger 4 |  |
| *DAAM1* | Disheveled-associated activator of morphogenesis 1 |  |
| *DAAM2* | Disheveled-associated activator of morphogenesis 2 |  |
| *DKK1* | Dickkopf WNT Signaling Pathway Inhibitor 1 |  |
| *DKK2* | Dickkopf-related protein 2 |  |
| *DKK4* | Dickkopf-related protein 4 |  |
| *DVL1* | Segment polarity protein dishevelled homolog DVL-1 |  |
| *DVL2* | Segment polarity protein dishevelled homolog DVL-2 |  |
| *DVL3* | Segment polarity protein dishevelled homolog DVL-3 |  |
| *EP300* | E1A binding protein p300 |  |
| *FBXW11* | F-Bpx And WD Repeat Domain Containing 11, | SCF ? |
| *FOSL1* | FOS-like Antigen 1 | FRA1 |
| *FRAT1* | Frequently Rearranged in Advanced T-Cell Lymphomas 1 |  |
| *FRAT2* | Frequently Rearranged in Advanced T-Cell Lymphomas 2 |  |
| *FZD1* | Frizzled -1 | FzE1 |
| *FZD10* | Frizzled-10 | FzE7; CD350 |
| *FZD2* | Frizzled-2 |  |
| *FZD3* | Frizzled-3 |  |
| *FZD4* | Frizzled-4 | FzE4 |
| *FZD5* | Frizzled-5 | FzE5 |
| *FZD6* | Frizzled-6 |  |
| *FZD7* | Fizzled-7 | FzE3 |
| *FZD8* | Frizzled-8 |  |
| *FZD9* | Frizzled-9 | FzE6 |
| *GPC4* | Glypican-4 |  |
| *GSK3B* | Glycogen synthase kinase-3 beta |  |
| *INVS* | Inversin | NPHP2 |
| *JUN* | Jun Proto-Oncogene |  |
| *LEF1* | Lymphoid enhancer binding factor 1 |  |
| *LRP5* | Low-density lipoprotein receptor-related protein 5 | LR3; LRP7 |
| *LRP6* | Low-density lipoprotein receptor-related protein 6 |  |
| *MAP3K7* | Mitogen-activated protein kinase kinase kinase 7 | TAK1; TGF1a |
| *MAPK10* | Mitogen-activated protein kinase 10 | JNK3; SAPK1b |
| *MAPK8* | Mitogen-activated protein kinase 8 | JNK; JNK1; SAPK1 |
| *MAPK9* | Mitogen-activated protein kinae 9 |  |
| *MMP7* | Matrix Metallopeptidase 7 | MPSL1; PUMP-1 |
| *MYC* | v-myc myelocytomatosis viral oncogene homolog |  |
| *NFATC1* | Nuclear factor of activated T-cells, cytoplasmic 1 | NFAT2; NFATC |
| *NFATC2* | Nuclear factor of activated T-cells, cytoplasmic 2 | NFAT1; NFATP |
| *NFATC3* | Nuclear factor of activated T-cells, cytoplasmic 3 | NFATx |
| *NFATC4* | Nuclear factor of activated T-cells, cytoplasmic 4 | NFAT3 |
| *NKD1* | Protein naked cuticle homology-1 |  |
| *NKD2* | Protein naked cuticle homology-2 |  |
| *NLK* | Serine/Threonine-protein kinase NLK |  |
| *NOTUM* | Palmitoleoyl-protein carboxylesterase NOTUM |  |
| *PLCB1* | Phospholipase C, Beta 1 | EIEE12, PLC154A, PLCB1B |
| *PLCB2* | Phospholipase C, Beta 2 | PLC-B2 |
| *PLCB3* | Phospholipase C, Beta 3 (Phosphatidylinositol-Specific) |  |
| *PLCB4* | Phospholipase C, Beta 4 | ARCND2, PI-PLC |
| *PORCN* | Porcupine homolog | DHOF, FODH, MG61, PORC, PNN |
| *PPARD* | Peroxisome proliferator acivated receptor delta | FAAR, NR1C2, NUC1, NUCI, PPARB |
| *PPP3CA* | Protein Phosphatase 3, catalytic subunit, alpha | CALN, CALNA, CALNA1, CCN1,CNA1, PPP2B |
| *PPP3CB* | Protein Phosphatase 3, catalytic subunit, beta | CALNA2, CALNB, CNA2, PP2Bbeta |
| *PPP3CC* | Protein Phosphatase 3, catalytic subunit, gamma | CALNA3, CNA3, PP2Bgamma |
| *PPP3R1* | Protein Phosphatase 3, regulatory subunit B, alpha | CALNB1, CNB, CNB1 |
| *PPP3R2* | Protein Phosphatase 3, regulatory subunit B, beta | CaNB2, CnB2 |
| *PRICKLE1* | Prickle planar cell politarity protein 1 | EMP1B, RILP |
| *PRICKLE2* | Prickle planar cell politarity protein 2 | EPM5 |
| *PRKACA* | Protein kinasae cAMP-activated catalytic subunit alpha | PKACA, PPNAD4 |
| *PRKACB* | Protein kinasae cAMP-activated catalytic subunit beta | PKA C-beta, PKACB |
| *PRKACG* | Protein kinasae cAMP-activated catalytic subunit gamma | KAPG, PKACg, BDPLT19 |
| *PRKCA* | Protein Kinase C alpha | AAG6, PKC-alpha PKCA, PRKACA |
| *PRKCB* | Protein Kinase C beta | PKC-beta, PKCB1, RPRKCB2, PRKCB |
| *PRKCG* | Protein Kinase C gamma | PKC-gamma, PKCC, PKCG, SCA14 |
| *PSEN1* | Presenilin 1 | AD3, FAD, Ps-1, PS1, S182 |
| *RAC1* | Ras-related C3 botulinum toxin substrate 1 | MIG5, Rac-1, TC-25, p21-RAC1 |
| *RAC2* | Ras-related C3 botulinum toxin substrate 2 | EN-7, p21-Rac2, Gx, HSPC022 |
| *RAC3* | Ras-related C3 botulinum toxin substrate 3 |  |
| *RBX1* | Ring-Box 1, E3 Ubiquitin Protein Ligase | RNF75, ROC1, BA554C12.1 |
| *RHOA* | Ras Homolog Family Member A | ARHA; RHOH12 |
| *ROCK2* | Rho-Associated, Coiled-Coil Containing Protein Kinase |  |
| *RUVBL1* | RuvB like AAA ATPase 1 | ECP54, NMP238, PONTIN, RVB1, TIH1, TIP49, TIP49A |
| *SENP2* | SUMO1/sentrin/SMT3 specific peptidase 2 | AXAM2, SMT3IP2 |
| *SERPINF1* | Serpin family F member 1 | EPC-1, O16, PEDF, PIG35 |
| *SFRP1* | Secreted Frizzled-Related Protein 1 | FRP, FRP1, FrzA, SARP2 |
| *SFRP2* | Secreted Frizzled-Related Protein 2 | FRP2, SARP1, SDF-5 |
| *SFRP4* | Secreted Frizzled-Related Protein 4 | FRPHE; sFRP-4 |
| *SFRP5* | Secreted Frizzled-Related Protein 5 | SARP3; FRP-1b; sFRP-5 |
| *SIAH1* | siah E3 ubiquitin protein ligase 1 | SIAH1A |
| *SKP1* | S-phase kinase-associated protein 1 | EMC19, p19A, TCEB1L, OCP2A |
| *SMAD3* | SMAD family member 3 | MADH3; MAD |
| *SMAD4* | SMAD family member 4 | MADH4; DPC4, |
| *SOST* | Sclerostin | CDD, DAND61, VBCH |
| *SOX17* | SRY-box 17 | VUR3 |
| *TBL1X* | Transducin beta like 1X-linked | EB1, SMAP55, TBL1 |
| *TBL1XR1* | Transducin beta like 1X-linked receptor 1 | C21, DC42, IRA1, MDR41, TBLR1 |
| *TBL1Y* | Transducin beta like 1, Y-linked | TBL1 |
| *TCF7* | Transcription Factor 7 | TCF-1 |
| *TCF7L1* | Transcritpion Factor 7-like 1 | TCF3 |
| *TCF7L2* | Transcritpion Factor 7-like 2 | TCF4 |
| *TP53* | tumor protein 53 | BCC7, LFS1, P53, TRP53 |
| *VANGL1* | VANGL planar cell polarity protein 1 | LLP2; STB2; STBM2; KITENIN |
| *VANGL2* | VANGL planar cell polarity protein 2 | LPP1; LTAP; STB1; STBM1 |
| *WIF1* | Wnt inhibitory factor 1 |  |
| *WNT1* | Wingless-type MMTV Integration Site Family, Member 1 |  |
| *WNT10A* | Wingless-type MMTV Integration Site Family, Member 10A |  |
| *WNT10B* | Wingless-type MMTV Integration Site Family, Member 10B |  |
| *WNT11* | Wingless-type MMTV Integration Site Family, Member 11 |  |
| *WNT16* | Wingless-type MMTV Integration Site Family, Member 16 |  |
| *WNT2* | Wingless-type MMTV Integration Site Family, Member 2 | IRP; INT1L1 |
| *WNT2B* | Wingless-Type MMTV Integration Site Family, Member 2B |  |
| *WNT3* | Wingless-type MMTV Integration Site Family, Member 3 |  |
| *WNT3A* | Wingless-type MMTV Integration Site Family, Member 3A |  |
| *WNT4* | Wingless-type MMTV Integration Site Family, Member 4 |  |
| *WNT5A* | Wingless-Type MMTV Integration Site Family, Member 5A |  |
| *WNT5B* | Wingless-type MMTV Integration Site Family, Member 5B |  |
| *WNT6* | Wingless-type MMTV Integration Site Family, Member 6 |  |
| *WNT7A* | Wingless-type MMTV Integration Site Family, Member 7A |  |
| *WNT7B* | Wingless-type MMTV Integration Site Family, Member 7B |  |
| *WNT8A* | Wingless-type MMTV Integration Site Family, Member 8A |  |
| *WNT8B* | Wingless-type MMTV Integration Site Family, Member 8B |  |
| *WNT9A* | Wingless-type MMTV Integration Site Family, Member 9A |  |
| *WNT9B* | Wingless-type MMTV Integration Site Family, Member 9B |  |
