# Supplementary material for: Expression of Wnt-signaling pathway genes and their associations with miRNAs in colorectal cancer
Source: Oncotarget. 2017 Dec 23;9(5):6075–85. doi: 10.18632/oncotarget.23636 (PMC5814196; doi:10.18632/oncotarget.23636)
Supplement: Supplementary file 4 [file oncotarget-09-6075-s004.docx]

| Supplemental Table 3: Wnt-Signaling Pathway genes with MSS tumors only | | | | | |
| --- | --- | --- | --- | --- | --- |
| Gene Name | Tumor Mean | Normal Mean | Fold Change | P-Value | Adjusted P-Value |
| *SFRP1* | 1.94 | 37.64 | 0.05 | 2.82E-52 | 7.77E-51 |
| *PPP3R2* | 0.21 | 0.93 | 0.23 | 4.56E-06 | 8.07E-06 |
| *CAMK2A* | 5.34 | 22.16 | 0.24 | 1.15E-35 | 1.32E-34 |
| *PRKCB* | 13.53 | 49.63 | 0.27 | 3.87E-37 | 5.34E-36 |
| *WNT5B* | 5.80 | 19.42 | 0.30 | 2.71E-33 | 2.20E-32 |
| *WNT1* | 0.34 | 1.13 | 0.30 | 7.53E-07 | 1.46E-06 |
| *MAPK10* | 9.11 | 27.60 | 0.33 | 1.54E-31 | 1.06E-30 |
| *WNT10B* | 1.22 | 3.54 | 0.34 | 1.43E-12 | 3.53E-12 |
| *WNT2B* | 21.00 | 54.20 | 0.39 | 4.89E-43 | 9.64E-42 |
| *PRKACB* | 63.18 | 158.66 | 0.40 | 1.47E-38 | 2.26E-37 |
| *WNT9A* | 2.44 | 6.08 | 0.40 | 3.56E-14 | 9.82E-14 |
| *DAAM2* | 23.56 | 51.67 | 0.46 | 6.30E-33 | 4.83E-32 |
| *CAMK2B* | 1.20 | 2.53 | 0.48 | 3.91E-05 | 6.42E-05 |
| *NFATC1* | 10.80 | 22.32 | 0.48 | 6.38E-19 | 2.45E-18 |
| *TCF7L1* | 8.26 | 17.04 | 0.48 | 6.31E-19 | 2.45E-18 |
| *WNT4* | 3.99 | 7.32 | 0.55 | 5.12E-10 | 1.14E-09 |
| *SFRP5* | 0.65 | 1.17 | 0.55 | 8.14E-04 | 1.22E-03 |
| *TBL1Y* | 0.26 | 0.46 | 0.56 | 4.10E-02 | 5.19E-02 |
| *PLCB2* | 31.43 | 56.12 | 0.56 | 3.04E-16 | 9.33E-16 |
| *RAC2* | 20.46 | 36.16 | 0.57 | 6.93E-16 | 2.08E-15 |
| *PRICKLE2* | 30.54 | 50.79 | 0.60 | 5.34E-17 | 1.68E-16 |
| *PRKACG* | 0.20 | 0.33 | 0.61 | 1.39E-02 | 1.92E-02 |
| *SOX17* | 1.21 | 1.98 | 0.61 | 3.75E-04 | 5.81E-04 |
| *CAMK2D* | 83.89 | 136.67 | 0.61 | 4.68E-31 | 2.93E-30 |
| *WNT10A* | 4.79 | 7.72 | 0.62 | 1.32E-04 | 2.14E-04 |
| *WNT7A* | 0.35 | 0.55 | 0.64 | 1.05E-01 | 1.25E-01 |
| *APC2* | 19.77 | 30.25 | 0.65 | 6.87E-13 | 1.76E-12 |
| *TCF7L2* | 122.11 | 180.80 | 0.68 | 2.80E-22 | 1.33E-21 |
| *WNT8B* | 1.78 | 2.61 | 0.68 | 2.75E-03 | 3.96E-03 |
| *SMAD4* | 82.35 | 117.11 | 0.70 | 5.10E-22 | 2.27E-21 |
| *WNT16* | 0.83 | 1.16 | 0.72 | 7.23E-02 | 8.83E-02 |
| *NFATC2* | 63.20 | 85.82 | 0.74 | 3.29E-07 | 6.67E-07 |
| *PSEN1* | 84.23 | 113.84 | 0.74 | 7.40E-19 | 2.76E-18 |
| *APC* | 97.41 | 131.11 | 0.74 | 7.28E-18 | 2.51E-17 |
| *PRICKLE1* | 7.36 | 9.89 | 0.74 | 1.08E-03 | 1.60E-03 |
| *FZD5* | 224.62 | 294.15 | 0.76 | 8.58E-13 | 2.15E-12 |
| *SFRP2* | 25.56 | 33.30 | 0.77 | 4.56E-02 | 5.72E-02 |
| *FRAT1* | 6.55 | 8.47 | 0.77 | 2.27E-03 | 3.34E-03 |
| *WNT9B* | 0.72 | 0.90 | 0.80 | 2.70E-01 | 3.08E-01 |
| *PPP3CC* | 16.60 | 20.57 | 0.81 | 6.02E-04 | 9.13E-04 |
| *PPARD* | 59.66 | 73.05 | 0.82 | 2.56E-06 | 4.65E-06 |
| *PRKCA* | 69.76 | 84.27 | 0.83 | 1.07E-07 | 2.26E-07 |
| *SMAD3* | 104.97 | 124.23 | 0.85 | 3.52E-06 | 6.31E-06 |
| *CCND3* | 34.40 | 40.58 | 0.85 | 1.57E-04 | 2.52E-04 |
| *CAMK2G* | 74.60 | 87.28 | 0.85 | 2.47E-06 | 4.54E-06 |
| *EP300* | 289.16 | 337.06 | 0.86 | 9.02E-14 | 2.44E-13 |
| *RBX1* | 20.09 | 23.14 | 0.87 | 2.75E-03 | 3.96E-03 |
| *CTBP1* | 164.68 | 188.31 | 0.87 | 1.38E-07 | 2.88E-07 |
| *PRKACA* | 53.66 | 60.68 | 0.88 | 3.21E-05 | 5.33E-05 |
| *WNT6* | 0.68 | 0.76 | 0.90 | 6.23E-01 | 6.51E-01 |
| *NFATC4* | 28.12 | 31.12 | 0.90 | 5.27E-02 | 6.55E-02 |
| *FZD1* | 25.54 | 27.90 | 0.92 | 1.03E-01 | 1.24E-01 |
| *DAAM1* | 52.54 | 56.63 | 0.93 | 9.01E-02 | 1.09E-01 |
| *PPP3CB* | 53.74 | 56.85 | 0.95 | 1.86E-01 | 2.14E-01 |
| *CTBP2* | 147.89 | 154.70 | 0.96 | 1.17E-01 | 1.38E-01 |
| *SIAH1* | 44.15 | 45.94 | 0.96 | 2.78E-01 | 3.15E-01 |
| *SERPINF1* | 30.19 | 31.40 | 0.96 | 5.20E-01 | 5.52E-01 |
| *CREBBP* | 266.24 | 273.55 | 0.97 | 1.70E-01 | 1.98E-01 |
| *PPP3CA* | 76.38 | 77.67 | 0.98 | 6.86E-01 | 7.06E-01 |
| *INVS* | 50.90 | 51.13 | 1.00 | 9.18E-01 | 9.21E-01 |
| *BTRC* | 40.02 | 39.85 | 1.00 | 9.21E-01 | 9.21E-01 |
| *NFATC3* | 159.07 | 157.77 | 1.01 | 7.57E-01 | 7.74E-01 |
| *MAPK8* | 60.07 | 59.48 | 1.01 | 7.92E-01 | 8.04E-01 |
| *NLK* | 48.15 | 46.84 | 1.03 | 4.80E-01 | 5.18E-01 |
| *FRAT2* | 23.37 | 22.71 | 1.03 | 5.42E-01 | 5.71E-01 |
| *MAPK9* | 64.79 | 62.72 | 1.03 | 3.81E-01 | 4.21E-01 |
| *TCF7* | 97.67 | 93.94 | 1.04 | 3.39E-01 | 3.77E-01 |
| *DVL2* | 29.20 | 28.05 | 1.04 | 3.96E-01 | 4.34E-01 |
| *JUN* | 229.02 | 218.97 | 1.05 | 3.18E-01 | 3.57E-01 |
| *CHD8* | 149.44 | 142.03 | 1.05 | 3.10E-02 | 4.04E-02 |
| *VANGL2* | 35.71 | 33.79 | 1.06 | 4.73E-01 | 5.14E-01 |
| *DVL3* | 148.78 | 140.59 | 1.06 | 1.49E-02 | 2.03E-02 |
| *PLCB3* | 97.66 | 92.20 | 1.06 | 1.23E-01 | 1.43E-01 |
| *FZD4* | 44.08 | 40.52 | 1.09 | 5.58E-02 | 6.87E-02 |
| *FBXW11* | 69.85 | 62.77 | 1.11 | 4.30E-03 | 6.12E-03 |
| *FZD9* | 0.41 | 0.36 | 1.12 | 6.47E-01 | 6.72E-01 |
| *CSNK1A1* | 288.43 | 256.04 | 1.13 | 5.27E-06 | 9.20E-06 |
| *SKP1* | 90.60 | 80.36 | 1.13 | 3.12E-04 | 4.89E-04 |
| *CSNK2B* | 79.09 | 69.69 | 1.13 | 1.78E-04 | 2.83E-04 |
| *WNT3A* | 0.39 | 0.34 | 1.16 | 4.88E-01 | 5.22E-01 |
| *LRP5* | 190.29 | 162.40 | 1.17 | 5.91E-07 | 1.16E-06 |
| *MAP3K7* | 72.57 | 61.36 | 1.18 | 1.06E-06 | 2.03E-06 |
| *FZD8* | 15.94 | 13.41 | 1.19 | 2.47E-02 | 3.28E-02 |
| *DVL1* | 71.57 | 59.85 | 1.20 | 1.76E-06 | 3.28E-06 |
| *SENP2* | 60.31 | 49.91 | 1.21 | 1.23E-06 | 2.32E-06 |
| *LRP6* | 164.21 | 135.32 | 1.21 | 6.94E-11 | 1.62E-10 |
| *VANGL1* | 63.18 | 52.01 | 1.21 | 1.68E-05 | 2.83E-05 |
| *CSNK1E* | 170.61 | 140.22 | 1.22 | 1.45E-14 | 4.18E-14 |
| *GSK3B* | 168.73 | 138.19 | 1.22 | 1.49E-13 | 3.89E-13 |
| *PORCN* | 9.09 | 7.36 | 1.23 | 1.82E-02 | 2.46E-02 |
| *AXIN1* | 101.07 | 79.99 | 1.26 | 4.15E-10 | 9.39E-10 |
| *CTNNBIP1* | 27.00 | 21.24 | 1.27 | 1.34E-05 | 2.28E-05 |
| *TBL1XR1* | 372.91 | 293.05 | 1.27 | 1.36E-15 | 3.99E-15 |
| *CXXC4* | 11.75 | 9.12 | 1.29 | 8.21E-03 | 1.14E-02 |
| *RAC3* | 2.65 | 2.02 | 1.31 | 2.77E-02 | 3.64E-02 |
| *FZD2* | 1.94 | 1.46 | 1.33 | 2.39E-02 | 3.20E-02 |
| *RHOA* | 277.99 | 207.18 | 1.34 | 3.09E-22 | 1.42E-21 |
| *FZD7* | 19.63 | 14.16 | 1.39 | 7.13E-06 | 1.23E-05 |
| *CUL1* | 75.17 | 53.39 | 1.41 | 2.91E-17 | 9.33E-17 |
| *PPP3R1* | 87.18 | 60.51 | 1.44 | 5.70E-18 | 2.02E-17 |
| *PRKCG* | 2.86 | 1.97 | 1.45 | 3.54E-02 | 4.53E-02 |
| *RAC1* | 185.06 | 126.22 | 1.47 | 2.06E-26 | 1.24E-25 |
| *TBL1X* | 49.35 | 33.39 | 1.48 | 2.22E-09 | 4.87E-09 |
| *FZD6* | 34.49 | 22.45 | 1.54 | 1.39E-13 | 3.69E-13 |
| *CSNK2A1* | 140.42 | 91.21 | 1.54 | 2.78E-23 | 1.42E-22 |
| *CCND2* | 768.38 | 475.09 | 1.62 | 1.55E-14 | 4.35E-14 |
| *CTNNB1* | 598.28 | 357.40 | 1.67 | 1.56E-41 | 2.69E-40 |
| *ROCK2* | 463.82 | 276.83 | 1.68 | 8.16E-35 | 8.05E-34 |
| *TP53* | 99.81 | 57.76 | 1.73 | 1.16E-19 | 4.73E-19 |
| *CACYBP* | 36.09 | 20.18 | 1.79 | 1.74E-23 | 9.23E-23 |
| *DKK1* | 0.83 | 0.44 | 1.87 | 3.53E-02 | 4.53E-02 |
| *RUVBL1* | 46.08 | 24.05 | 1.92 | 8.44E-34 | 7.77E-33 |
| *BAMBI* | 9.79 | 5.02 | 1.95 | 3.04E-07 | 6.25E-07 |
| *WNT5A* | 60.97 | 30.65 | 1.99 | 1.06E-17 | 3.57E-17 |
| *GPC4* | 56.20 | 28.07 | 2.00 | 4.54E-24 | 2.50E-23 |
| *CSNK2A1P* | 2.34 | 1.15 | 2.04 | 7.23E-11 | 1.66E-10 |
| *PLCB1* | 47.96 | 23.20 | 2.07 | 4.07E-18 | 1.48E-17 |
| *FZD3* | 43.14 | 20.47 | 2.11 | 1.20E-21 | 5.17E-21 |
| *PLCB4* | 355.00 | 164.12 | 2.16 | 2.92E-20 | 1.22E-19 |
| *CSNK2A2* | 49.08 | 22.30 | 2.20 | 4.24E-32 | 3.08E-31 |
| *WNT7B* | 0.98 | 0.44 | 2.21 | 4.89E-04 | 7.49E-04 |
| *DKK4* | 0.95 | 0.38 | 2.48 | 6.04E-03 | 8.51E-03 |
| *CCND1* | 308.59 | 118.05 | 2.61 | 4.70E-56 | 2.16E-54 |
| *LEF1* | 41.90 | 13.03 | 3.21 | 8.92E-36 | 1.12E-34 |
| *WNT3* | 1.86 | 0.56 | 3.29 | 2.77E-12 | 6.60E-12 |
| *FOSL1* | 13.04 | 3.78 | 3.45 | 2.01E-17 | 6.62E-17 |
| *MYC* | 192.41 | 49.64 | 3.88 | 2.13E-49 | 4.90E-48 |
| *FZD10* | 5.58 | 1.37 | 4.08 | 3.06E-09 | 6.60E-09 |
| *DKK2* | 5.42 | 1.22 | 4.46 | 2.44E-12 | 5.91E-12 |
| *AXIN2* | 371.67 | 78.65 | 4.73 | 2.35E-64 | 3.25E-62 |
| *WIF1* | 2.95 | 0.59 | 5.00 | 4.43E-07 | 8.85E-07 |
| *WNT11* | 18.98 | 3.46 | 5.49 | 3.97E-24 | 2.28E-23 |
| *NKD2* | 38.75 | 4.82 | 8.04 | 1.13E-52 | 3.90E-51 |
| *SFRP4* | 37.30 | 4.43 | 8.42 | 9.78E-34 | 8.43E-33 |
| *NKD1* | 183.29 | 18.34 | 9.99 | 1.39E-57 | 9.60E-56 |
| *WNT2* | 11.17 | 0.83 | 13.49 | 4.88E-35 | 5.18E-34 |
| *MMP7* | 17.19 | 1.13 | 15.26 | 1.22E-22 | 6.02E-22 |
| *NOTUM* | 16.12 | 0.62 | 25.97 | 3.16E-31 | 2.08E-30 |
